# Effects of Pressure to Abort on Women’s Emotional Responses and Mental Health

**DOI:** 10.7759/cureus.34456

**Published:** 2023-01-31

**Authors:** David C Reardon, Tessa Longbons

**Affiliations:** 1 Health Policy, Elliot Institute, St. Peters, USA; 2 Research, Charlotte Lozier Institute, Arlington, USA

**Keywords:** post-abortion mental health, post-abortion adjustments, health policy, pregnancy loss, unsafe abortions, reproductive rights, mental health, abortion

## Abstract

Background

Women who feel pressured to agree to abortion are more likely to experience negative emotional and mental health reactions. But relatively little research has been conducted to explore the types and degree of pressures women face and their associated effects. Our study aims to investigate five types of pressure women may face and a sample of effects that may be associated with unwanted abortions.

Methods

A retrospective survey was distributed through a marketing research firm and completed by 1000 females aged 41 to 45, inclusive, living in the United States. The survey instrument included demographic questions and analog scales for respondents to rate the pressure to abort arising from male partners, family members, other persons, financial concerns, and other circumstances and 10 variables related to both positive and negative outcomes.

Results

Among 226 respondents who reported a history of abortion, perceived pressure to abort was significantly associated with more negative emotions; more disruption of daily life, work, or relationships; more frequent thoughts, dreams, or flashbacks to the abortion; more frequent feelings of loss, grief or sadness about the abortion; more moral and maternal conflict over the abortion decision; a decline in overall mental health that they attribute to their abortions; more desire or need for help to cope with negative feelings about the abortion. Overall, 61% reported high levels of pressure on at least one scale. Women with a history of abortion were four times more likely to quit the survey than women who did not have abortions, and those with a history of feeling pressured to abort also reported higher levels of stress related to completing the survey.

Discussion

Perceived pressures to choose abortion should be assessed before an abortion to better guide risk assessments, decision-making, and analyses of post-abortion adjustments in light of these risk factors. A history of abortion, especially when there was pressure to abort, is associated with more stress completing questionnaires touching on abortion experiences and with a higher dropout rate, a finding that is consistent with the view that abortion surveys are likely to underrepresent the experiences of the women who experience the most stress and negative reactions to their abortions. Abortion providers should screen for perceived pressures to abort and be prepared to offer counseling and services that will help women to avoid unwanted abortions.

## Introduction

The 2008 literature review by the American Psychological Association’s Task Force on Mental Health and Abortion identified 15 risk factors for more negative mental health outcomes following abortion [1]. Among these is the “perceived pressure from others to terminate a pregnancy.” Other reviews and studies have also identified pressure to choose abortion as a risk factor for greater difficulty in coping with the subsequent abortion [2-6]. Yet there is a great diversity in the types of pressures women self-report [7-10]. Pressure from male partners, parents, employers, health care providers, sex traffickers, and other persons may have varying degrees of effects on both the abortion decision and subsequent adjustments [10-13]. Similarly, pressure from situational factors, such as financial pressure, maternal health issues, fetal malformation, and other circumstances may also have varying degrees of effect on coping and satisfaction with abortion [14].

While all of the above-named pressures to undergo abortions are well-known, relatively little research has been done to differentiate these pressures and their separate and cumulative effects on post-abortion mental health. In this exploratory study, we seek to (a) confirm or disprove the association between pressures to abort and more negative post-abortion adjustments, and (b) begin the process of identifying which pressures have the greatest negative effects on post-abortion adjustments.

## Materials and methods

Study design and setting

This study is a retrospective survey of American women who completed an electronic survey form in October of 2022. The study design was approved by the Sterling Institutional Review Board (approval no. 10225). The survey instrument was developed in consultation with experts in abortion counseling and researchers who have published in the field of abortion’s association with emotional and mental health effects. The survey included five statements regarding pressures to abort and 10 statements regarding outcome variables, collectively shown in Table 1, along with the abbreviation for each pressure stated in this report. Respondents indicated their responses using a slider on a visual analog scale displayed on their own electronic devices. While no numbers were shown to the respondents when they slid their markers, their responses on the visual analog scale were automatically converted to the appropriate percentage in a range from 0 to 100.

**Table 1:** Survey questions and abbreviations

Abbreviation	Complete statement or question	Scale of Agreement (0 to 100)
MalePr	I felt pressure to abort from my male partner.	Not at all | Very much so
FamilyPr	I felt pressure to abort from one or more family members.	Not at all | Very much so
OtherPr	I felt pressure to abort from someone else.	Not at all | Very much so
FinPr	I felt pressure to abort from financial concerns.	Not at all | Very much so
OtherCircPr	I felt pressure to abort from other circumstances.	Not at all | Very much so
PositiveEmotions	My positive emotions regarding the abortion are . . .	None at all | Very high
NegativeEmotions	My negative emotions regarding the abortion are . . .	None at all | Very high
InterferedwLife	Thoughts and feelings about my abortion have negatively interfered with daily life, work, or relationships.	Not at all true | Very true
NeededHelp	I have desired or needed help to better cope with negative feelings or behaviors due to my abortion.	Not at all true | Very true
IntrusiveThoughts	I have had frequent thoughts, dreams, or flashbacks to the abortion.	Not at all true | Very true
FrequentLoss	I have had frequent feelings of loss, grief, or sadness about the abortion.	Not at all true | Very true
BetterMentalHlth	Abortion made my mental health . . .	Very much worse | Very much better
SurveyStress	Completing this survey has increased feelings of stress.	Not at all true | Very true
MoralConflct	The idea of abortion conflicted with my moral beliefs.	Not at all | Very much so
MaternalConflict	The idea of abortion conflicted with my maternal desires.	Not at all | Very much so

In brief, respondents rated the level of pressure, if any, they experienced from their male partner, their family, other persons, financial pressures, and other circumstances. To further our analyses, we also constructed the average score (AvgPr) and the maximum score (MaxPr) each woman reported across each of these five scales. The outcome scales rated each respondent’s level of experience of positive emotions, negative emotions, disruption of normal life, desire for help to cope, intrusive thoughts, frequent feelings of loss, their assessment of abortion’s impact on their mental health, and whether completing the survey increased feelings of stress.

Population

The surveyed population was drawn from 28 million Cint panelists in the United States [15]. Cint panelists are persons who voluntarily complete surveys using their own electronic devices in exchange for small rewards. Our selection criteria required Cint to obtain 1,000 completed surveys from females who are residents of the United States who were 41 to 45 years of age, inclusive, at a cost of three dollars per completed survey. This narrow age range was chosen to eliminate the confounding effects of age while capturing the experience of women who have completed the majority of their reproductive lives.

## Results

A total of 1161 persons identified by Cint to be females aged 41 to 45 answered at least the first page of our survey. The first two pages contained only demographic questions which were used to disqualify 122 respondents whose self-reported age or gender was outside our limits. Of the remaining 1039 qualified respondents, 39 failed to complete the survey, yielding a 96% completion rate. Of these qualified respondents, 248 women reported a history of abortion of whom 226 completed the full survey, for a completion rate of 91%. Women with a history of abortion were over four times (odds ratio (OR)=4.43, 95% confidence interval (CI)=2.31-8.49) more likely to drop out of the survey at or after the first question related to abortion compared to women who did not report a history of abortion and were routed to a different set of questions regarding their reproductive lives.

Demographic characteristics are shown in Table 2. The first two columns allow a comparison of the U.S. census data for all persons over 18 years of age alongside the demographics for all 28 million U.S. residents in the Cint survey panels. The third column shows demographics provided by Cint for all women aged 41 to 45 in their survey panel. The fourth and fifth columns show the 1000 women who completed our survey, and the subset of 226 women who had abortions in our survey sample and report the demographics participants provided on the first page of our survey. The table reveals a reasonably good approximation of females in this age group relative to the national census data with four exceptions. First, U.S. census data shows that 11% of all residents over age 18 have not completed high school, whereas only 3% of the Cint panel of women 41 to 45 years of age have not completed high school. In large part, this may be due to the fact that middle-aged women have had more time to advance their education. In addition, less educated persons may be less inclined to agree to participate in survey panels. Second, our respondents somewhat underrepresent lower income groups, compared to both U.S. census data and all Cint panelists. Third, while the 226 women reporting a history of abortion in our panel are relatively similar to the entire Cint sample for women of this age group, national studies of abortion reveal that abortion rates among black women are three to four times higher than that of white women [16], a finding that is not reflected in our sample. Finally, our sample somewhat overrepresents women from the South U.S. census region.

**Table 2:** Percentage of demographic characteristics in the United States census, Cint national panel, survey sample, and the subgroup of respondents who had abortions All numbers are percentages for the associated demographic characteristic

	U.S. Census Data for All Persons	Cint U.S. Survey Panel for All Persons	Our Survey Sample (n=1000)	Our Abortion Subgroup (n=226)
Region				
Northeast	17	19	14	16
South	38	44	44	43
Midwest	21	19	21	19
West	24	18	21	22
Educational Attainment				
Less than high school graduate	11	4	4	1
High school graduate	26	27	33	35
University/Higher Education	49	54	46	49
Postgraduate Education	14	18	18	15
Household Income (2021)				
Under $25,000	17	23	13	12
$25,000-$49,999	19	17	15	14
$50,000-$79,999	19	24	26	28
$80,000-$99,999	9	11	19	19
Over $100,000	36	25	27	26
Ethnicity				
Asian	6	3	5	6
Black	14	13	15	14
Hispanic	19	12	14	15
White	59	68	59	57
Other	2	4	7	8

The distributions for all the scales used are shown in Table 3, including the means, standard deviation (SD), and quartiles. The quartiles for financial pressure, for example, show that 25% of respondents rated financial pressure as 15 or below, 50% (the median) rated this pressure as 63 or below, and 75% rated it as 85 or lower, with the last 25% rating it between 85 and 100. MaxPr shows over half of the women reporting at least one score above 91. Additional analyses of MaxPr revealed nearly one-third of the women (31.4%; 95% CI: 25.4% to 37.9%) rated at least one of the pressures at the extreme highest of the scale (100).

**Table 3:** Descriptive statistics of the distribution of responses for pressure scales The columns of this table represent for each question the number of respondents (N), the mean reported value, the standard deviation (SD), the minimum observed value (Min), the lower 25% quartile (25%), the median (50% quartile), the 75% percent quartile (75%), and the maximum (Max) observed value.

Abbreviation from Table 1	N	Mean	SD	Min	25%	Median	75%	Max
MalePr	226	31.3	35.4	0	0	12	63	100
FamilyPr	226	34.7	37.8	0	0	15	71	100
OtherPr	226	23.7	32.3	0	0	5.5	46	100
FinPr	226	54.6	36.6	0	15	63	85	100
OtherCircPr	226	64.7	33.5	0	47	73	98	100
MaxPr	226	80.3	25.7	0	69	91.5	100	100
AvgPr	226	41.8	21.9	0	27.8	40.1	56.8	100
PositiveEmotions	226	50.4	30.5	0	29	49	73	100
NegativeEmotions	226	50.7	33	0	23	51	78	100
InterferedwLife	226	35.7	33	0	3	30	59	100
NeededHelp	226	33.9	32.7	0	2	28	61	100
IntrusiveThoughts	226	33.2	33.6	0	1	22	60	100
FrequentLoss	226	39.3	34.5	0	3	35	67	100
BetterMentalHlth	226	49	23.4	0	35	50	61	100
SurveyStress	226	36.7	30.9	0	5	32.5	61	100
MoralConflict	226	49.1	34.8	0	19	50.5	76	100
MaternalConflict	226	46.3	35.1	0	9	50	76	100

**Figure 1:**
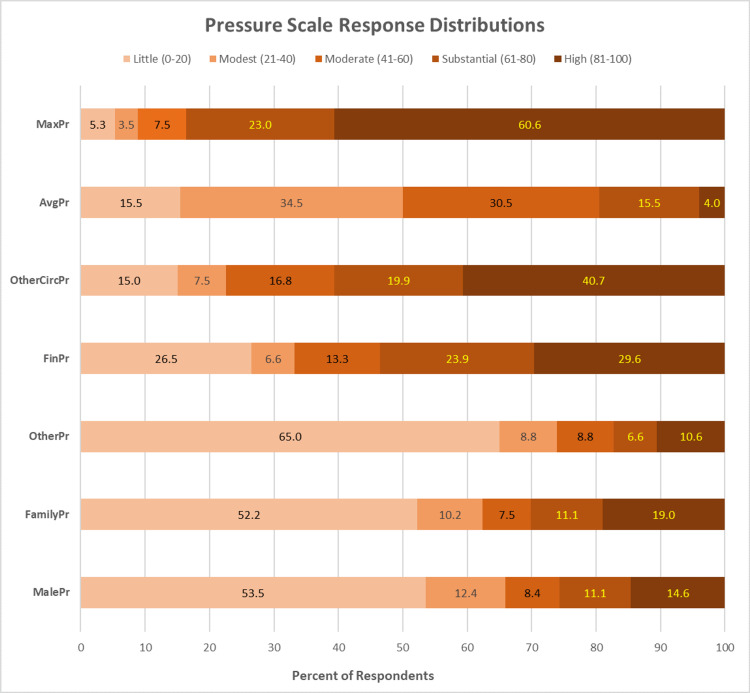
Pressure scale responses by the percentage of respondents grouped into five ranges Abbreviation of pressures taken from Table 1.

Figure 1 shows the percentage of respondents who reported little (<20 ), modest (21 to 40), moderate (41 to 60), substantial (61 to 80), and high (>80) levels of pressure for each pressure scale. For example, the MaxPr distribution using this scale revealed that 83.6% of women reported substantial to high levels of pressure on at least one scale.

Table 4 shows the correlations between our five pressure scales and 10 outcome scales. It reveals that the three interpersonal pressure scales (male partner, family, and other persons) are positively correlated to each other, indicating that many women report pressure from more than one other person. Feelings of pressure from financial concerns were mildly but significantly correlated to pressures from persons, but feelings of pressure from other circumstances were not. Notably, however, the mean score for pressure from other circumstances was the highest for all the means, yet at the same time this independent variable was the least strongly correlated to the outcome variables. By contrast, all three scales for pressure from other persons were significantly correlated with more negative outcomes on every outcome scale. Pressure from financial concerns was significantly correlated to worse outcomes for eight of the 10 outcome variables. Financial concerns were not correlated to intrusive thoughts or fewer positive feelings, though both might prove to be correlated with a larger sample size in light of the strongly skewed confidence intervals.

**Table 4:** Correlation matrix of pressure scales and outcome scales The mean (M) and standard deviations (SD) are shown for each variable.  Correlations between variables are shown along with the range in parentheses which shows the 95% confidence interval for each correlation.  * indicates p < .05. ** indicates p < .01.

Variable	M	SD	MalePr	FamilyPr	OtherPr	FinPre	OtherCircPr
1. MalePr	31.33	35.36					
2. FamilyPr	34.67	37.75	.36** (.24, .47)				
3. OtherPr	23.74	32.28	.42** (.31, .52)	.53** (.43, .62)			
4. FinPr	54.62	36.58	.19** (.06, .31)	.15* (.02, .28)	.17** (.05, .30)		
5. OtherCircPr	64.68	33.53	0.02 (-.11, .15)	0.0 (-.13, .13)	0.1 (-.03, .23)	.40** (.29, .51)	
6. PositiveEmotions	50.37	30.54	-0.13 (-.26, .00)	-0.07 (-.20, .06)	-0.08 (-.21, .05)	-0.11 (-.24, .02)	0.06 (-.07, .19)
7. NegativeEmotions	50.65	32.97	.40** (.29, .51)	.34** (.21, .45)	.36** (.25, .47)	.32** (.20, .43)	.16* (.03, .28)
8. InterferedwLife	35.71	32.98	.40** (.28, .50)	.32** (.20, .44)	.45** (.34, .55)	.14* (.01, .26)	0.08 (-.05, .21)
9. NeededHelp	33.95	32.72	.42** (.30, .52)	.40** (.29, .51)	.48** (.37, .57)	.16* (.03, .28)	0.1 (-.03, .22)
10. IntrusiveThoughts	33.2	33.61	.46** (.35, .56)	.36** (.24, .47)	.54** (.44, .62)	0.1 (-.04, .22)	0.12 (-.01, .25)
11. FrequentLoss	39.3	34.49	.41** (.30, .51)	.39** (.27, .50)	.46** (.35, .56)	.17* (.04, .29)	.15* (.02, .28)
12. BetterMentalHlth	49.03	23.37	-.17** (-.30, -.04)	-.19** (-.31, -.06)	-0.13 (-.25, .00)	-0.02 (-.15, .11)	-0.01 (-.14, .12)
13. SurveyStress	36.72	30.93	.42** (.30, .52)	.28** (.15, .39)	.28** (.16, .40)	.14* (.01, .27)	0.06 (-.07, .19)
14. MoralConflict	49.11	34.79	.40** (.29, .51)	.31** (.19, .42)	.40** (.29, .51)	.21** (.08, .33)	0.11 (-.02, .24)
15. MaternalConflict	46.32	35.08	.40** (.28, .50)	.32** (.20, .44)	.39** (.28, .50)	.24** (.11, .36)	0.11 (-.02, .23)

Correlations with the two constructed pressure scales, MaxPr and AvgPr, are shown in Table 5, along with correlations between each of the dependent variables. These results revealed that AvgPr provided a better correlation to outcome variables than MaxPr. In addition, negative outcomes generally showed moderate to strong correlations with each other, indicating that women who experienced one negative mental health outcome were more likely to experience negative outcomes across several domains.

**Table 5:** Correlations between the constructed pressure scales and the outcome scales The mean (M) and standard deviations (SD) are shown for each variable.  Correlations between variables are shown along with the range in parentheses which shows the 95% confidence interval for each correlation.  * indicates p < .05. ** indicates p < .01.

Variable	M	SD	1	2	3	4	5	6	7	8	9	10	11
1. AvgPr	41.81	21.89											
2. MaxPr	80.28	25.65	.55** (.46, .64)										
3. PositiveEmotions	50.37	30.54	-0.11 (-.23, .02)	-0.11 (-.24, .02)									
4. NegativeEmotions	50.65	32.97	.51** (.41, .60)	.29** (.17, .41)	-.53** (-.62, -.43)								
5. InterferedwLife	35.71	32.98	.44** (.33, .54)	.15* (.02, .27)	-.35** (-.46, -.23)	.62** (.53, .70)							
6. NeededHelp	33.95	32.72	.50** (.39, .59)	.19** (.07, .32)	-.31** (-.43, -.19)	.59** (.50, .67)	.84** (.79, .87)						
7. IntrusiveThoughts	33.2	33.61	.50** (.40, .59)	.26** (.14, .38)	-.30** (-.41, -.18)	.56** (.46, .64)	.68** (.60, .74)	.71** (.64, .77)					
8. FrequentLoss	39.3	34.49	.51** (.40, .60)	.30** (.17, .41)	-.29** (-.40, -.16)	.59** (.49, .66)	.67** (.59, .74)	.69** (.61, .75)	.80** (.75, .85)				
9. BetterMentalHlth	49.03	23.37	-.17* (-.29, -.04)	-.15* (-.27, -.02)	.56** (.47, .65)	-.47** (-.57, -.36)	-.45** (-.55, -.34)	-.43** (-.53, -.31)	-.39** (-.50, -.27)	-.47** (-.56, -.36)			
10. SurveyStress	36.72	30.93	.38** (.26, .49)	.16* (.03, .29)	-.37** (-.47, -.25)	.55** (.45, .63)	.58** (.49, .66)	.53** (.43, .62)	.54** (.44, .63)	.54** (.44, .63)	-.32** (-.43, -.20)		
11. MoralConflict	49.11	34.79	.46** (.35, .56)	.24** (.11, .36)	-.32** (-.44, -.20)	.67** (.59, .73)	.61** (.52, .69)	.57** (.48, .65)	.48** (.37, .58)	.58** (.48, .66)	-.30** (-.41, -.17)	.47** (.36, .57)	
12. MaternalConflict	46.32	35.08	.47** (.36, .56)	.22** (.09, .34)	-.29** (-.41, -.17)	.64** (.55, .71)	.61** (.52, .68)	.52** (.42, .61)	.48** (.37, .57)	.61** (.52, .69)	-.33** (-.44, -.20)	.41** (.29, .51)	.70** (.62, .76)

## Discussion

Our findings confirmed that women who perceived pressure to abort, especially from their male partners, families, or other persons, are more likely to report more negative reactions to abortion. Those experiencing pressure reported more negative emotions; more disruption of daily life, work, or relationships; more frequent thoughts, dreams, or flashbacks to the abortion; more frequent feelings of loss, grief, or sadness about the abortion; more moral and maternal conflict over the abortion decision; a decline in overall mental health that they attribute to their abortions; and more desire or need for help to cope with negative feelings about the abortion. In addition, women who reported feeling more pressure to choose abortion also reported higher levels of stress completing the survey. This last finding is consistent with previous studies suggesting that questionnaire-based studies of abortion and mental health are likely to underreport negative reactions due to self-censure bias [3].

In our sample, 61% of the women reported experiencing a high level of pressure to abort on at least one scale. However, the scale with the highest mean score was for pressure to abort from other circumstances, OtherCircPr, which was the one scale that was also the least correlated to any of the outcome scales. Given that this open-ended category had the highest average intensity, it suggests that there are a number of additional types of pressure that are most important in the abortion decision of many women. Future research efforts should incorporate more detailed scales examining all the many reasons why women choose abortion, including health concerns for themselves or for fetal malformation, having already reached their family size goals, instability in the relationship with the male partner, and conflicts with short-term and/or long-term life goals, for example [17].

In addition, we found that women with a history of abortion were more likely to drop out of the survey once the topic of abortion was raised. Among those who completed the survey, those who reported feeling pressured to abort experienced more stress completing the survey than those who faced little or no pressure to abort. These findings underscore the fact that every survey of women’s abortion experiences is likely to suffer from selection bias, with women who feel the most pressure to abort and who are most likely to have negative reactions being least likely to participate in or complete follow-up surveys.

It is unclear how well our survey sample reflects the national population of all women who have had an abortion. Our sample is clearly limited to residents in the United States and our age range of women aged 41 to 45 years. Moreover, the demographic characteristics shown in Table 2 suggest our findings may underrepresent lower-income and lower-educated women and some minority groups, at least in comparison to nationally reported abortion rates. Therefore, caution should be exercised in drawing any conclusions regarding the actual frequency of women feeling pressured to abort in the general population. On the other hand, it is highly likely that the correlations between the types of pressures identified, and the negative outcome variables utilized in our study, do apply to the general population of women who have experienced abortion. In that regard, our study shows that it is important for future studies on emotional responses to abortion to include questions rating the level of pressures women face, particularly from other people, prior to undergoing their abortions, as these pressures are clearly important risk factors for more negative outcomes.

Another weakness in our study is that the 10 outcome scales utilized in our survey were entirely self-assessments. We have no data on psychiatric diagnoses nor did we use any psychometric scales. The latter was not employed since these would have vastly lengthened the survey, depressed response rates and to the degree that they are often limited to feelings within the last week or 30 days, and may have failed to represent the “entire history” of women’s post-abortion adjustments. In that regard, while we would encourage the use of psychometric scales in future investigations, we believe the 10 self-assessment scales used in the present study provide an important contribution to our understanding of how various pressures to abort impact different aspects of post-abortion adjustments.

An additional weakness is that our data is based entirely on retrospective ratings. Memories of past events may be colored by years of reflection, subsequent experiences, and reaction formation. Clearly, it would be better to gather information about the types and degrees of pressures women face to have an abortion during the counseling period prior to an abortion. Identification of these risk factors would provide an opportunity for better counseling and discussion of these pressures and their associated risks. It would also provide better data for correlation to post-abortion adjustment data collected in subsequent case series investigations.

## Conclusions

Women frequently choose abortion due to perceived pressures from other people, financial concerns, or other circumstantial pressures. These pressures, individually and/or together, are strongly associated with more negative emotions about their abortion; more disruptions of their daily life, work, or relationships; more frequent dreams, flashbacks, or intrusive thoughts about their abortions; more frequent feelings of loss, grief, or sadness about their abortions; more moral and maternal conflict over their abortion decisions; a perceived decline in their overall mental health that they attribute to their abortions; and a higher degree of desire or need for help to cope with negative feelings about their abortions. 

Additional research is needed to better identify the types of pressures women face and the variety of outcomes associated with each type of pressure. Abortion providers should screen for perceived pressures to abort and should counsel women accordingly. Therapists and counselors offering care to those struggling with post-abortion emotional adjustments or mental health issues should also assess perceived pressures to abort.
